# Leukemia Inhibitory Factor Downregulates Human Papillomavirus-16 Oncogene Expression and Inhibits the Proliferation of Cervical Carcinoma Cells

**DOI:** 10.1155/2011/463081

**Published:** 2011-06-04

**Authors:** Joseph M. Bay, Bruce K. Patterson, Nelson N. H. Teng

**Affiliations:** ^1^Division of Gynecologic Oncology, Department of Obstetrics and Gynecology, Stanford University School of Medicine, 300 Pasteur Drive, Stanford CA 94305-5317, USA; ^2^Department of Pathology, Stanford University School of Medicine, 300 Pasteur Drive, Stanford CA 94305-5317, USA

## Abstract

The constitutive proliferation and resistance to differentiation and apoptosis of neoplastic cervical cells depend on sustained expression of human papillomavirus oncogenes. Inhibition of these oncogenes is a goal for the prevention of progression of HPV-induced neoplasias to cervical cancer. SiHa cervical cancer cells were transfected with an HPV-16 promoter reporter construct and treated with leukemia inhibitory factor (LIF), a human cytokine of the interleukin 6 superfamily. SiHa and CaSki cervical cancer cells were also assessed for proliferation by MTT precipitation, programmed cell death by flow cytometry, and HPV E6 and E7 expression by real-time PCR. LIF-treated cervical cancer cells showed significantly reduced HPV LCR activation, reduced levels of E6 and E7 mRNA, and reduced proliferation. We report the novel use of LIF to inhibit viral oncogene expression in cervical cancer cells, with concomitant reduction in proliferation suggesting re-engagement of cell-cycle regulation.

## 1. Introduction

Human Papillomavirus (HPV) is a necessary causative agent for cervical cancer [1, 2], the second most common cancer in women [3] and third overall cause of cancer mortality worldwide [4]. HPV has also been detected in anal cancer [5], cancers of the head and neck, particularly the oropharynx and larynx [5, 6], and there is evidence that some breast cancers contain HPV genomes [7]. At present, no approved, effective nonsurgical intervention for cervical dysplasia or for the underlying HPV infection exists. For localized and advanced localized cervical carcinoma, the concurrent administration of chemotherapy and radiation has been successful, if significantly toxic, but the treatment of recurrent and metastatic disease remains a challenge. For metastatic cervical cancer, standard chemotherapy is generally palliative rather than curative, and there is limited experience with biologics in this group of patients; therefore, a new treatment modality is clearly needed. With over 13,000 cases of invasive cervical cancer, 50,000 cases of carcinoma *in situ*, and as many as 1,000,00 cases of cervical dysplasia diagnosed each year in the US alone, and with the majority of spending going to followup and treatment of neoplasia, a noninvasive treatment would have a tremendous effect on both women's health and the financial burden of HPV. 

The expression of the viral gene products E6 and E7, regulated by the viral long control region (LCR), is also predictive of progression toward cancer: higher levels of viral mRNA correlate with higher-grade lesions [8]. E6 [9] and E7 [9, 10] have also been shown to contribute to the invasiveness and metastatic aggressiveness [10, 11] of transformed cells, and the targeted inhibition by 50%–90% *in vitro* of HPV oncoproteins (using siRNA [12], peptide aptamers [13], or a transfected E2 gene [14]) typically results in p53 reactivation and HPV-specific cell-cycle exit and/or apoptosis [15]. Furthermore, both radiation [16] and many forms of chemotherapy [17] more effectively kill cervical cancer cells in which E6 and/or E7 expression is attenuated. 

We have identified an endogenous human cytokine, LIF, which is capable of directly and drastically repressing E6 and E7 expression in cultured cervical squamous carcinoma cells and reducing or halting proliferation of such cells. As a phase I-tested[18] pharmaceutical with little toxicity, LIF is a promising candidate for the treatment of CIN, cervical carcinoma, and other HPV-dependent pathologies, either independently or in combination with cytotoxic chemotherapy.

## 2. Materials and Methods

### 2.1. Cell Culture

Human cervical cancer lines CaSki and SiHa were purchased from the American Type Culture Collection and cultured in Dulbecco's Modified Eagle Medium (DMEM) supplemented with 100 IU/mL penicillin, 100 *μ*g/mL streptomycin, and (except where noted) 10% fetal calf serum at 5% CO_2_. In several assays, where noted, serum was reduced or removed entirely.

### 2.2. Drugs

LIF (Millipore) was stored in phosphate-buffered saline (PBS) at 4°C and diluted in DMEM prior to use. TNFa, IL-6, and EGF (Biosource, Invitrogen) were dissolved in PBS with BSA, stored at −20°C, and diluted in DMEM or RPMI prior to use.

### 2.3. Generation of an HPV-16 Reporter Cell Line

The cervical cancer cell line SiHa was chosen because of its relatively high native HPV-16 LCR activity and significant expression of the E6 and E7 genes, similar to that in a progressing dysplastic lesion. The cells were transfected with the plasmid pLCRGLuc, which contains a complete HPV-16 LCR cloned upstream of a secreted *Gaussia* luciferase gene as well as a neomycin resistance gene driven by an SV40 promoter. Cells were selected with 400 **μ**g/mL G418 for seven days.

### 2.4. Cell Proliferation Assay

Cell proliferation was determined by CellTiter 96 (Promega) (3-(4,5-Dimethylthiazol-2-yl)-2,5-diphenyltetrazolium bromide (MTT) assay. Cells were seeded at 5,000 per well in a 96-well plate and treated with the specified cytokines. After the indicated periods, the cells were incubated according to the manufacturer's protocol with the MTT labeling solution for 4 h, then with the solubilization/stop solution overnight. Reduction of MTT to MTT-formazan, indicating cellular metabolic activity, was quantified by measurement of absorbance at 570 nm on a SpectraMax microplate reader (Molecular Devices) and background absorbance of 650 nm was subtracted. All experiments were done in triplicate or higher multiplicate for statistical power.

### 2.5. Luciferase Assays

For firefly luciferase, cells were seeded in 12-well plates at a density of 50,000/well and transfected with the indicated reporter plasmids using SuperFect (QIAGEN, Alameda, Calif, USA) according to manufacturer's instructions. After 18 hours, medium was replaced and the cells treated with the indicated stimuli for the time periods noted. Luciferase was released from cells using the supplied lysis buffer and luminescence measured using luciferase substrate (New England Biolabs, Ipswich, Mass, USA).

For *Gaussia* luciferase, SiHa-LCR-gLuc cells were seeded in 96-well plates at a density of ~5,000/well and treated as indicated. Following incubation, 20 (micro)L of supernatant from each well was transferred to a new plate. *Gaussia* luciferase reagent (New England Biolabs) was added and the luminescence measured for 5 seconds.

### 2.6. Quantitative Real-Time PCR

In order to directly measure the expression of E6 and E7 mRNA, we designed primer sets and probes specific to the E6 gene and the E7 gene; oligonucleotides were synthesized by Eurofins MWG Operon (Huntsville, AL). Primer sequences for the E6 mRNA were 5′-CAAACCGTTGTGTGATTTGTTAATTA-3′ and 5′-GCTTTTTGTCCAGATGTCTTTGC-3′ and the probe was 5′[6-FAM]TGTATTAACTGTCAAAAGCCACTGTGTCCTGAAGAA[TAMRA-6-FAM]-3′, corresponding to nucleotides 382-444. For E7, primer sequences were 5′-GTGACTCTACGCTTCGGTTGT-3′ and 5′-GCCCATTAACAGGTGTTCCA-3′ and the probe sequence was 5′[6-FAM]CGTACAAAGCACACACGTAGACATTCGTAC[BHQ1a-6FAM]-3′, corresponding to nucleotides 743–794 (or 743–1955 in the SiHa variant). Following experimental treatments, RNA was harvested using the RNeasy kit (Qiagen), quantified by spectrophotometry (NanoDrop 8000, Thermo Scientific), equal quantities amplified using the TaqMan kit (Applied Biosystems) according to the standard protocol (qv) on an ABI Prism 7700. We estimated the quantity of HPV mRNA relative to *β*-actin mRNA using the 2^−ΔΔCT^ method [19].

### 2.7. Intracellular Phosphospecific Flow Cytometry

Trypsinized cells (10,000 for each timepoint) were fixed with 1.2% paraformaldehyde at room temperature for 10 minutes, rinsed with PBS containing 1% BSA, permeabilized in PBS/90% ice-cold methanol, and stored at −20°C overnight or for up to 2 weeks. Prior to staining, cells were washed twice in PBS containing 1% BSA. Cells were stained on ice with the indicated antibodies for 30 minutes at 4°C and analyzed on a FC500 flow cytometer (Beckman-Coulter). Further analysis was performed using cytobank (http://www.cytobank.org/).

### 2.8. Statistics

Statistical analysis (unpaired *t*-test) was performed using GraphPad software (graphpad.com) as needed. *P* values indicating statistical significance are represented by a single (*P* < .1) or double asterisk (*P* < .05) on the figures.

## 3. Results and Discussion

### 3.1. LIF Inhibits HPV-16 LCR-Driven Transcription

**Figure 1:**
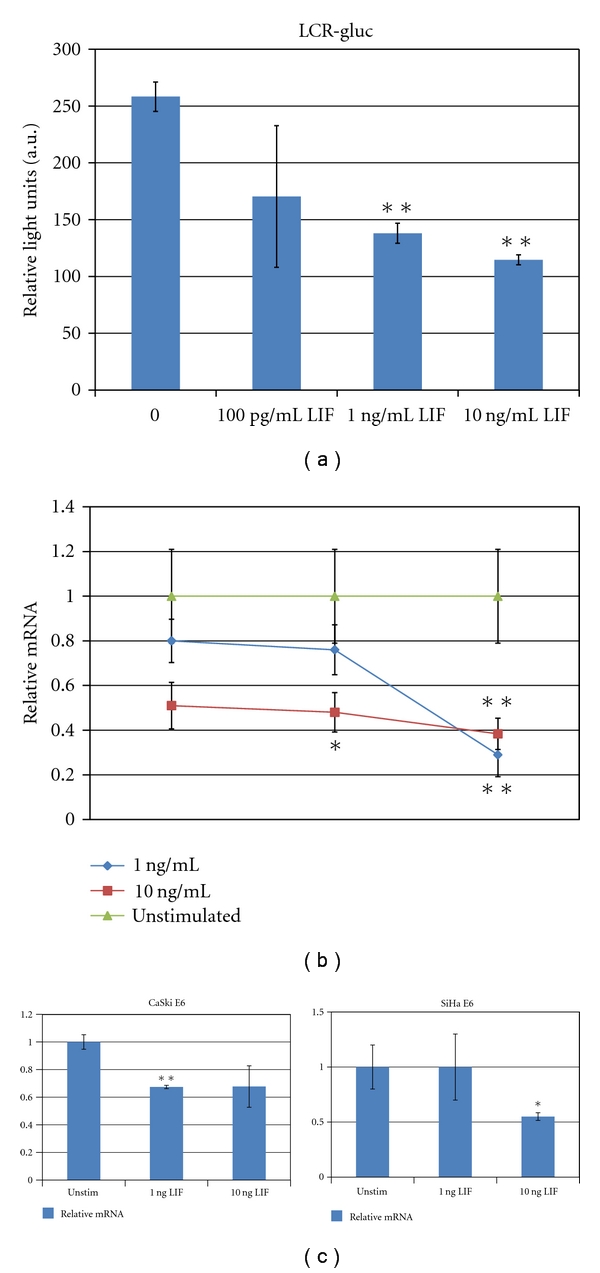
(a) Luciferase expression in LIF-treated and untreated SiHa pGLuc cells. (b) CaSki cells were treated with the indicated concentrations of LIF for 24 hours. Quantitative real-time PCR was performed as described. Error bars represent standard error of the mean. (c) CaSki and SiHa cells were treated with the indicated concentrations of LIF for 72 hours. Quantitation of E6 relative to *β*-actin is represented in arbitrary units. Error bars represent standard error of the mean. Asterisks represent significance (*P < .1, **P < .05).

In this study, we compared the transcriptional activity of the HPV-16 LCR in untreated cervical SiHa cells and cells treated with various cytokines. The IL-6 superfamily member LIF reduced HPV-16 LCR expression by approximately 60% in a time- and dose-dependent manner (Figure 1(a)). In order to verify that the observed decrease in LCR transcription is functionally significant and not an artifact of the reporter system, we examined the effect of LIF on mRNA expression by quantitative real-time PCR. CaSki cervical cancer cells were treated with 1 ng/mL or 10 ng/mL of LIF or with PBS for one, two, or three days. The expression of E7 mRNA was significantly reduced over this time course, initially in a dose-dependent manner (Figure 1(b)), although at three days, cells treated with the low or high concentrations of LIF showed similar inhibition of E7; E6 inhibition followed a similar trend (Figure 1(c)).

### 3.2. Induction of Phosphorylation of STAT3 In Cervical Cancer Cells

**Figure 2:**
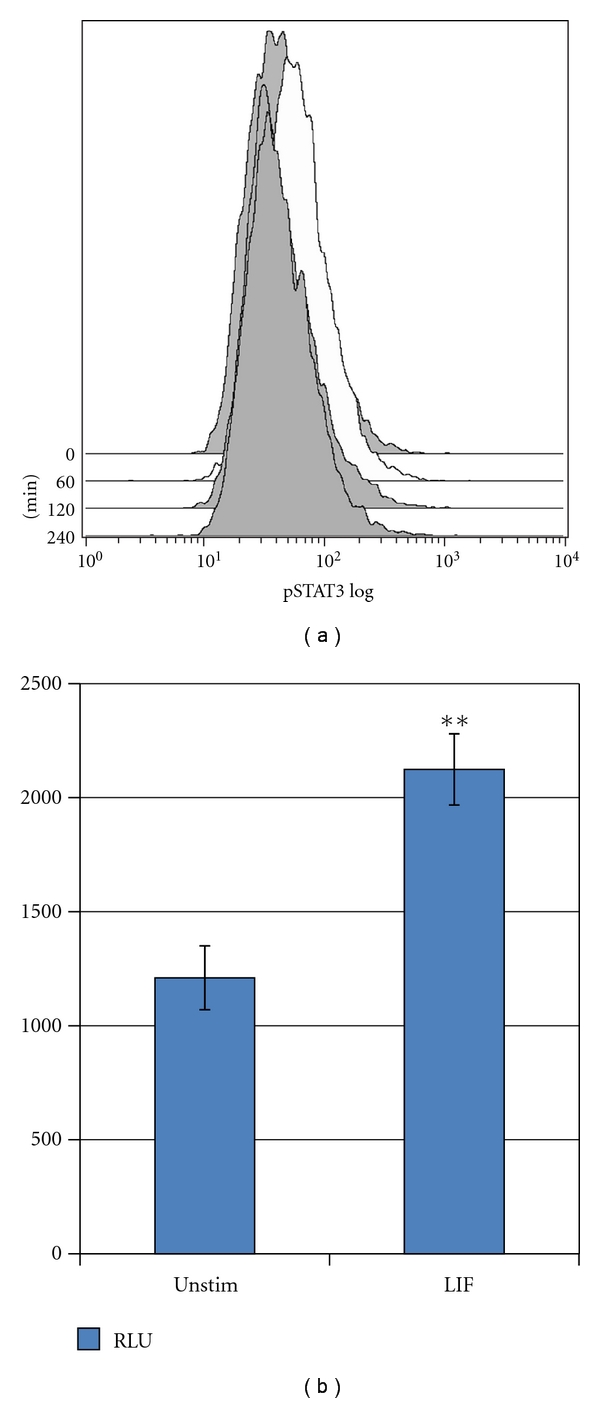
(a) SiHa cells were stimulated with LIF (10 ng/mL) for the times indicated (in minutes), and the level of phospho-STAT3(y705) measured. STAT3 is transiently phosphorylated following stimulation with LIF within 60 minutes, returning to baseline level by 120 minutes. Histograms are colored according to the log_10_-fold increase in mean fluorescence intensity relative to unstimulated cells. (b) CaSki cells transfected with a STAT3 reporter plasmid were treated with 50 ng/mL LIF for 6 hours prior to assay. The increase in relative light units is shown. Error bars represent standard error of the mean. Asterisks represent significance (**P < .05).

Because LIF is known to activate members of the JAK-STAT pathway, typically JAK1 and STAT3 in a cell-specific manner through binding to the heterodimeric LIFR-gp130 receptor [19, 20], we evaluated the effect of LIF on STAT3 activation in SiHa cells. STAT3 is a multifunctional signaling molecule generally considered to promote survival, proliferation, and tumorigenesis but can also be involved in the initiation of senescence and programmed cell death [21]. Following treatment with LIF, STAT3 was phosphorylated on tyrosine residue 705, indicative of activation, in a transient fashion (Figure 2(a)). 

To determine if the observed phosphorylation was accompanied by transcriptional activation, we used the reporter plasmid 4xM67 pTATA TK-Luc; SiHa cells were transfected; after 18 hours, cells were treated with 10 ng/mL LIF or PBS. After six hours, cells were lysed and analyzed as described. The activity of the reporter plasmid was 1.8-fold higher in LIF-treated cells than in untreated cells (Figure 2(b)).

### 3.3. Proliferation of HPV-Transformed Cells

**Figure 3:**
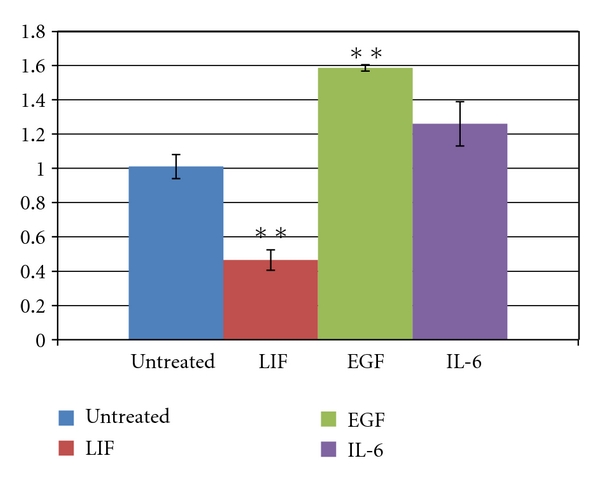
CaSki cells grown for 40 hours in the presence of the indicated agents were assayed by MTT for proliferation/metabolic activity. EGF and IL-6 enhanced cell number, while LIF inhibited proliferation. Error bars represent standard error of the mean. Asterisks represent significance (**P < .05).

CaSki cells were seeded in 96-well plates at a density of ~5000/well in growth medium. After the cells attached (~6 hours), the medium was replaced with serum-free medium containing LIF, the LCR-inhibiting cytokines IL-6 or EGF [22], or PBS. After 40 hours the proliferation of cells was measured using the MTT assay described earlier. As expected, EGF promoted the proliferation of the cells relative to untreated cells. The LIF-treated cells, however, proliferated much less rapidly, only reaching a final population density approximately half that of untreated cells (Figure 3).

Treatment of HPV-16-transformed cervical cancer cells with LIF inhibits the viral LCR, causing a substantial decrease in the abundance of the E6/E7 mRNA. This inhibition is concomitant with reduced proliferation. The absence of a significant loss of membrane asymmetry (data not shown) leads us to reason that LIF-treated cells undergo cell-cycle arrest rather than apoptosis. Whether this is specific to the cell lines used or would generally be the case in HPV-transformed cells remains to be seen. 

It has been postulated that high-level transcription of HPV is normally repressed by a cytokine-mediated intercellular control mechanism and that disruption of this is necessary for malignant transformation [23]. Native or exogenous cytokines, by activating or inactivating signaling pathways subverted by HPV, could allow the cell to revert from the malignant state back toward differentiation or programmed cell death. Several cytokines, including TGF-*β*1 and IL-4, are known to modulate transcription of HPV at the level of the LCR though in some cases, this is insufficient for reversal of the transformed phenotype. While IL-6 exposure represses the transcriptional activity of the HPV LCR [24], it nonetheless induces increased proliferation in both normal and cancerous cervical cells [25]. Exposure to TGF-*β*1, however, results in the downregulation of HPV oncogenes [26] and inhibits growth of early but not late HPV-immortalized cells [27]. Notably, TGF-*β*1 fails to induce the downregulation of E6 and E7 in SiHa cells and does not cause these cells to arrest. The interferons, particularly IFN-a2 and IFN-g, are potent inhibitors of HPV and have displayed some promise as therapeutics for HPV infection, but the actual clinical effectiveness has been inconsistent. Interferon-responsive elements which enhance transcriptional activity have been identified in the LCR [28], and late passage or malignantly transformed cells generally do not respond to IFN-*γ* [29] or other inhibitory cytokines [23].

LIF's role in promoting the differentiation of lymphocytes into anti-inflammatory regulatory cells [30] may be cause for concern, as the presence of such immunoregulatory cells has been found to support tumor tolerance and progression, contributing to migration and metastasis of tumor cells [31]. 

However, this potential increase in immune evasion is likely outweighed by the loss of viral oncogene expression. Additionally, LIF has demonstrated potent anti-HIV activity [32], most likely due to its role as a suppressor of inflammation, suggesting its use in HIV-seropositive CIN and cervical cancer patients would have multiple therapeutic benefits.

## 4. Conclusions

Figure 1 shows the effect of LIF on the expression of the HPV LCR and on two oncogenes controlled by this promoter. In two unrelated cell lines, both HPV-16 transformed, LCR-dependent transcription was significantly inhibited by LIF. Other means of achieving similar reductions in HPV mRNA, such as siRNA or peptide aptamer transfection, are observed to be sufficient to inhibit the growth of cervical cancer cells *in vitro* and *in vivo.* As shown in Figure 2, LIF increases the activity of the already constitutively active STAT3 in cervical cancer cells. While the significance of this activation is as yet uncertain, the STAT3 pathway appears to be involved in HPV-16 pathogenesis [33] and may be a target for therapeutic intervention. LIF strongly inhibits the growth of HPV-16 transformed cervical cells and appears to act at least in part through an HPV-specific mechanism.
